# Acute myocardial infarction caused by tumor embolus originating from upper tract urothelial carcinoma: a case report

**DOI:** 10.1186/s40959-020-00073-9

**Published:** 2020-09-07

**Authors:** Taku Yasui, Yohei Okuda, Wataru Shioyama, Toru Oka, Tatsuya Nishikawa, Risa Kamada, Koji Hatano, Kazuo Nishimura, Masashi Fujita

**Affiliations:** 1 Department of Onco-Cardiology, Osaka International Cancer Institute, 3-1-69 Otemae, Chuo-ku, Osaka, 541-8567 Japan; 2 Department of Urology, Osaka International Cancer Institute, Osaka, Japan

**Keywords:** Paradoxical embolism, Patent foramen ovale, Neoplasm, Aspiration, Coronary occlusion

## Abstract

Coronary emboli from malignant tumors rarely cause acute myocardial infarction. We report the case of a patient with tumor embolism from an upper tract urothelial carcinoma that caused acute myocardial infarction via a patent foramen ovale. Coronary blood flow was restored by embolus aspiration without stenting. Clinicians must consider malignant tumor embolism as a possible cause of acute myocardial infarction.

## Introduction

Tumor embolus is rare cause of acute myocardial infarction (AMI). Lung carcinoma was the most common source of coronary malignant tumor emboli, which was caused by direct tumor invasion to pulmonary veins and left atrium [1]. Here, we describe AMI caused by tumor embolus in a patient with upper tract urothelial carcinoma (UTUC).

## Case presentation

**Fig. 1:**
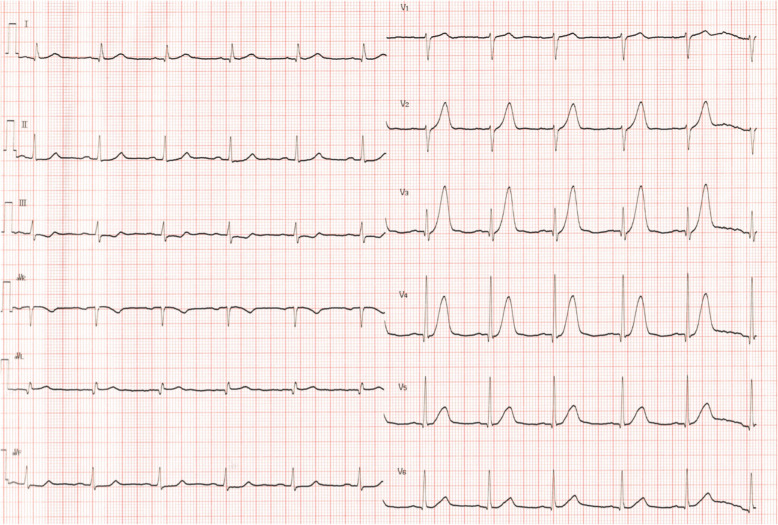
Twelve-lead electrocardiogram on admission. The electrocardiogram demonstrates hyperacute T waves in V2–4

**Fig. 2:**
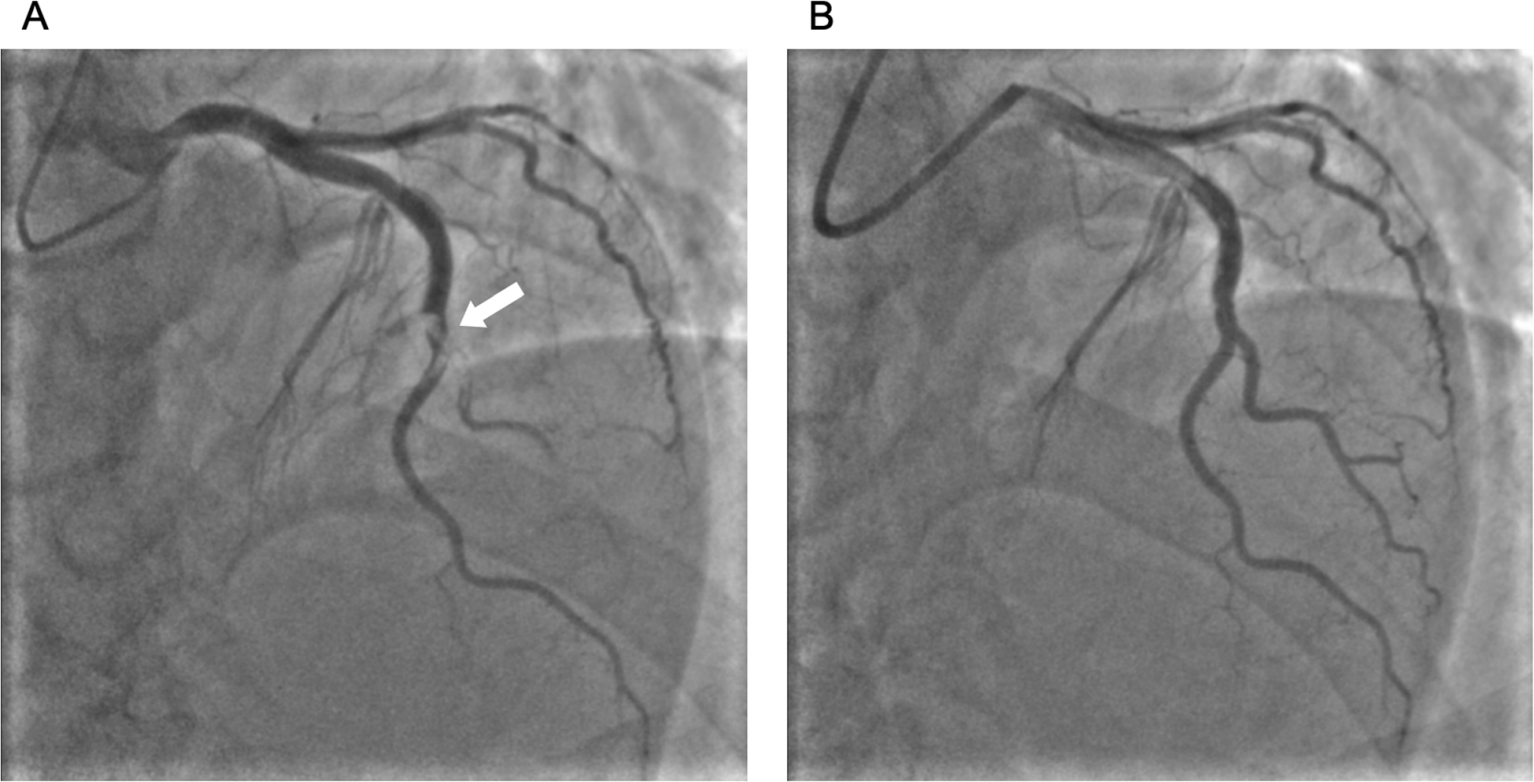
Left coronary angiography. **a** Initial left coronary angiography of cranial view revealing embolic obstruction straddling the bifurcation in the left anterior descending artery and diagonal branch (arrow). **b** Left coronary angiography after aspiration showing the restoration of blood flow

**Fig. 3:**
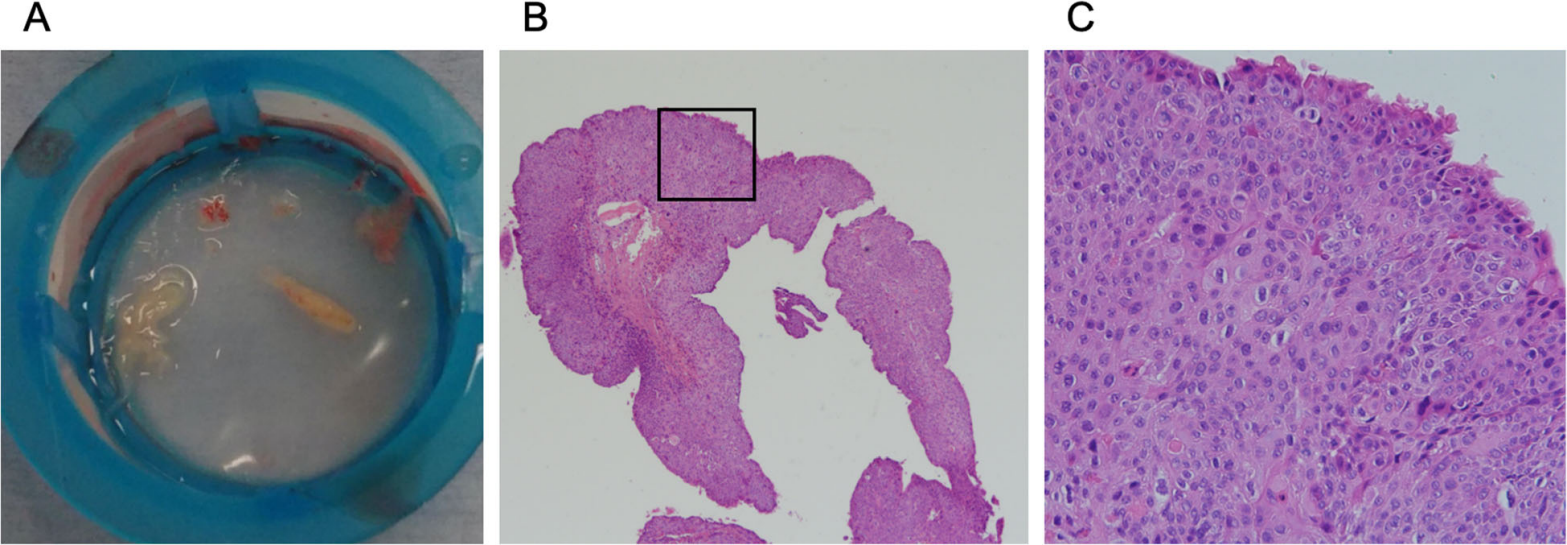
Histopathological findings. **a** Retrieved embolus. **b, c** Hematoxylin and eosin staining revealed atypical urothelial cell proliferation

**Fig. 4:**
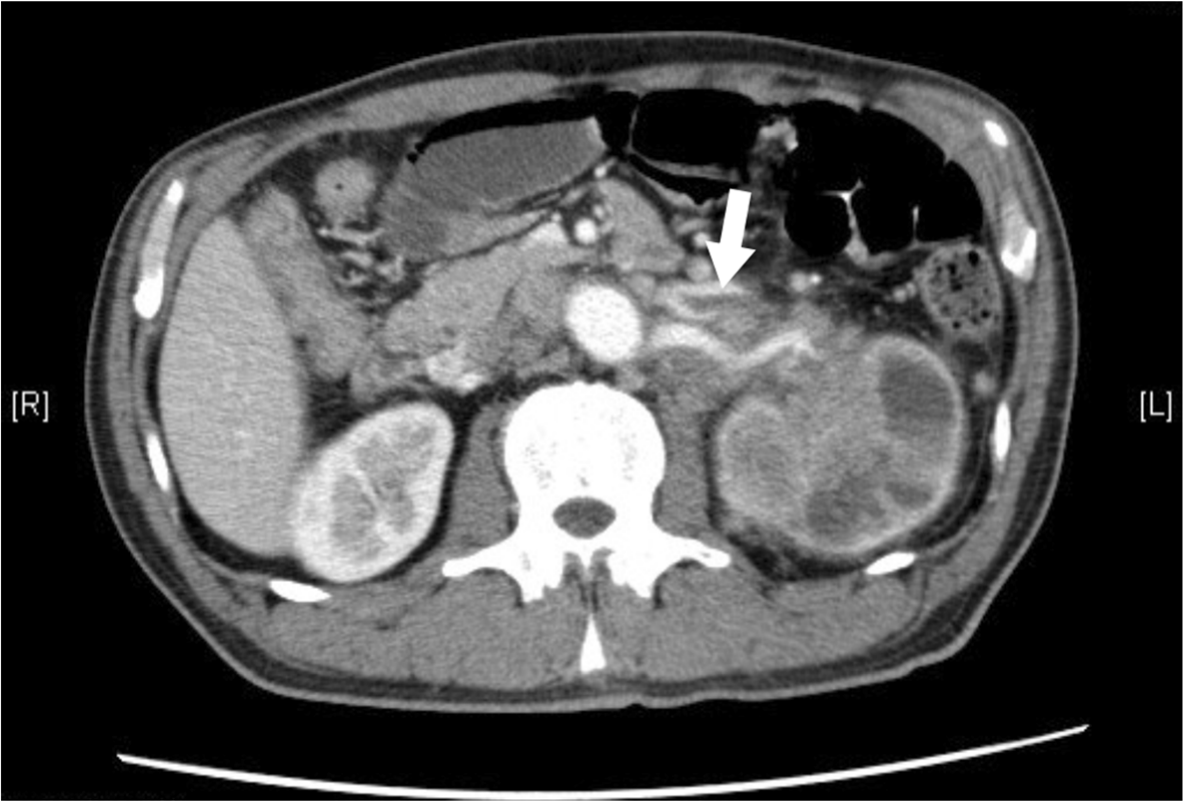
Contrast-enhanced computed tomography showed that tumor invading the left renal vein (arrow)

**Fig. 5:**
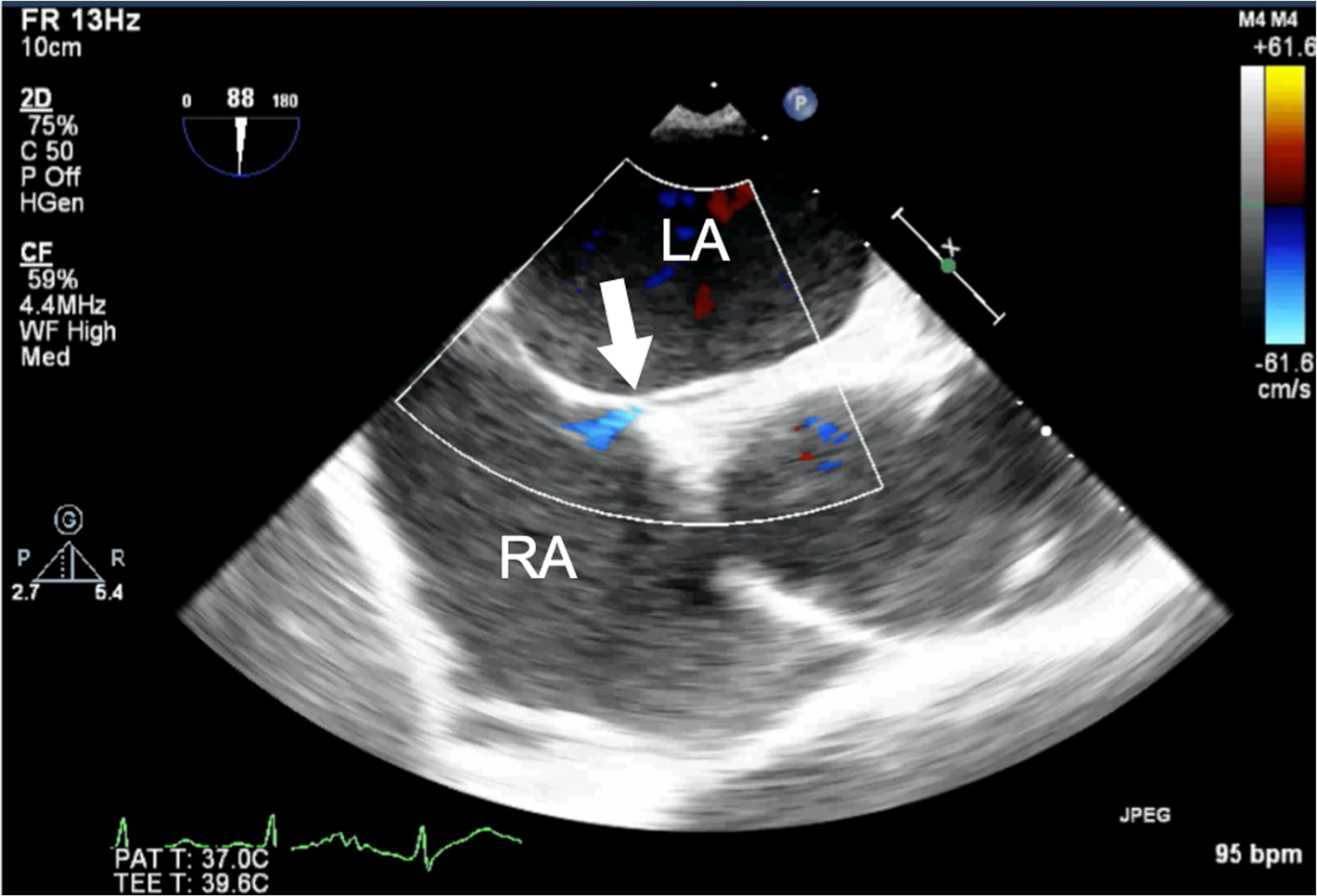
Transesophageal echocardiography showing a patent foramen ovale and shunt flow (arrow). LA: left atrium, RA: right atrium

**Fig. 6:**
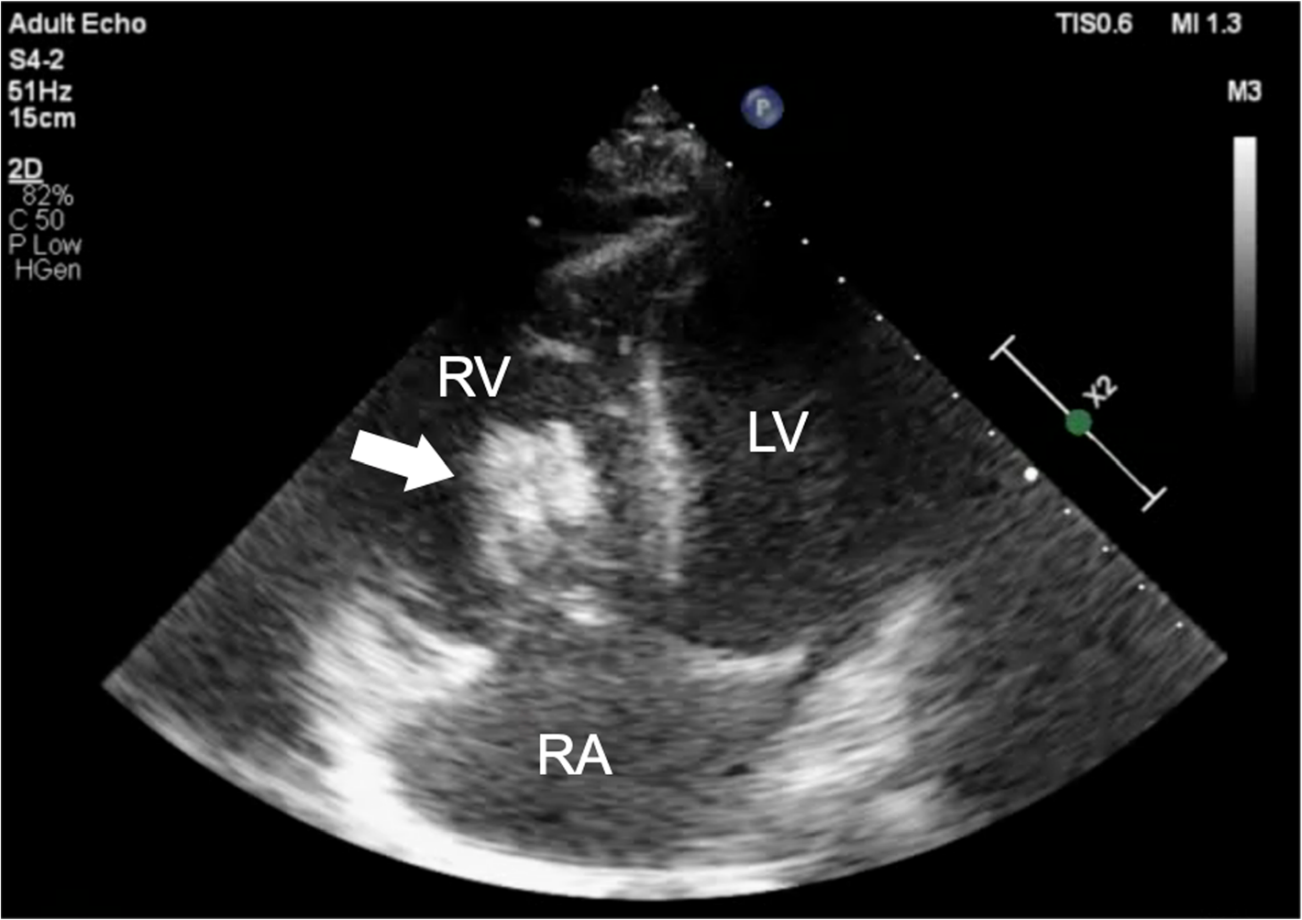
Transthoracic echocardiography on readmission showing right ventricular dilatation and a mobile mass attached to the tricuspid valve (arrow). RV: right ventricle, LV: left ventricle, RA: right atrium

A 62-year-old man diagnosed with UTUC and paraaortic lymph node metastasis (T4N2M0) was admitted to our hospital 2 h after sudden onset of severe chest pain after 2 cycles of chemotherapy with cisplatin and gemcitabine. On admission, his blood pressure was 120/81 mmHg, heart rate was 88 beats/min, respiratory rate was 20 breaths/min, and oxygen saturation was 96% on room air. He had no history of cardiovascular disease and no conventional coronary risk factors, including hypertension, diabetes mellitus, dyslipidemia, and cigarette smoking. Investigations performed at the time of admission revealed the following: creatine kinase (45 U/L; normal range, 59–248 U/L), creatine kinase-MB (1.0 ng/mL; normal range, < 5 ng/mL), and no elevated levels of cardiac troponin I (0.023 ng/mL; normal range, ≤0.026 ng/mL). The D-dimer level was elevated (3.6 μg/mL; normal range, 0–1.0 μg/mL). Contrast-enhanced computed tomography (CT) showed no pulmonary embolism and no aortic dissection. A 12-lead electrocardiography showed hyperacute T waves in V2–4 leads (Fig. 1). Transthoracic echocardiography showed akinesis of the left ventricular anteroseptal-apical wall. He was diagnosed with AMI and emergency coronary angiography (CAG) was performed. CAG revealed emboli straddling the bifurcation between the left anterior descending artery and diagonal artery (Fig. 2a). Aspiration was performed with a Thrombuster II catheter (KANEKA Medix Corporation, Osaka, Japan) retrieving a substantial amount of organized material, after which Thrombolysis in Myocardial Infarction (TIMI) grade 3 flow was achieved (Fig. 2b). An intravascular ultrasound revealed no evidence of atherosclerotic plaques, and hence, we finished the procedure without stenting. Histopathological analysis revealed that the emboli originated from an UTUC (Fig. 3a-c). Contrast-enhanced CT showed tumor invasion to the left renal vein (Fig. 4) and no lung metastasis. To examine the intracardiac shunt, we performed transesophageal echocardiography, which confirmed a patent foramen ovale (PFO) (Fig. 5). The maximum elevated serum levels of creatine kinase and creatine kinase-MB at following 9 h after admission were 1371 U/L and 137.1 ng/mL, respectively, which declined during the following days. He was discharged from our hospital after 10 days after an uneventful course of admission. The patient was readmitted to our hospital due to worsening of dyspnea 45 days after discharge. Transthoracic echocardiography showed right ventricular dilatation and a mobile mass attached to the tricuspid valve (Fig. 6), which suggested that the dyspnea was caused by a pulmonary artery embolism. On the second day, he developed progressive hypoxemia and systemic hypotension, leading to death.

## Discussion

This report presents a case of a patient with AMI caused by coronary tumor embolism. Tumor emboli originated from an UTUC invading the left renal vein. Paradoxical coronary embolism through the PFO was thought to be the mechanism of AMI. Aspiration of the tumor embolus without stenting restored coronary blood flow.

Coronary embolism is the underlying cause of 2.9% of AMI [2]. Embolus in coronary artery is commonly thromboembolus. Coronary tumor emboli, especially malignant tumor emboli, are very rare. Of the 147 patients with coronary embolism, two patients (1.4%) had malignancy as the etiology for coronary embolism [3]. In patients with malignant coronary tumor embolism, lung carcinoma was the most common source of tumor embolus because lung carcinoma can directly invade the pulmonary vein and left atrium [1]. Lung metastasis could cause coronary tumor embolism via the same mechanism. To the best of our knowledge, this is the first case report of coronary embolism caused by an UTUC. Contrast-enhanced CT showed no evidence of lung metastasis into the pulmonary vein in the patient. Clinicians should thus pay attention to the possibility of coronary tumor embolism in patients with malignancies other than lung tumors.

PFO is the most common congenital heart abnormality. A previous autopsy-based study reported that the prevalence of PFO was about 27% [4]. About half of patients with cryptogenic stroke, which accounts for approximately 40% of ischemic strokes, have a PFO [5]. Paradoxical embolism via PFO is a likely mechanism for stroke in this population. On the other hand, paradoxical embolism is a rare cause of AMI. Previous study reported that there were 33 (0.51%) presumed paradoxical coronary embolisms among 6502 patients with AMI [6]. The mechanism of the low incidence of AMI is not well understood; however, blood flow distribution could contribute to the low incidence of AMI. In our case, transesophageal echocardiography revealed a PFO. In addition, contrast-enhanced CT showed tumor invasion of the left renal vein but no lung metastasis. Taken together, we believe that paradoxical embolism caused AMI in our patient.

PFO closure should be considered for secondary prevention of paradoxical embolism. Several studies showed that percutaneous PFO closure significantly reduced the risk of recurrent stroke [7–9]. However, patients aged > 60 years were excluded from these studies. In addition, the efficacy and the safety of PFO closure in patients with advanced cancer remain unclear. For these reasons, we did not perform PFO closure in this patient.

If there is angiographic evidence of coronary embolism, embolus aspiration should be considered. Although routine thrombus aspiration for AMI does not appear to improve clinical outcomes [10], embolus aspiration could restore coronary blood flow and may assist in the determination of the origin of the embolus by pathological examination [11]. In our case, coronary blood flow was restored by aspiration without stenting because CAG and intravascular ultrasound revealed no evidence of atherosclerotic plaques. Histopathological examination revealed that the aspirated material was a tumor embolus originating from UTUC.

Cisplatin-based chemotherapy is associated with an increased risk of thromboembolic events [12]. In our case, tumor embolus was the exact cause of AMI, which was revealed by the histopathological examination. On the other hand, the origin of the pulmonary emboli was unknown because the autopsy of this patient was not carried out. Although the size and echotexture of the mass attached to the tricuspid valve indicated that pulmonary emboli were tumor emboli, thromboemboli might partially contribute to pulmonary embolism.

In conclusion, malignant tumor embolus can induce AMI in a patient with upper UTUC without lung metastasis. Although it is a rare cause of AMI, we must be aware of it.

## Data Availability

The datasets used and/or analysed during the current study are available from the corresponding author on reasonable request.

## Abbreviations

AMI: Acute myocardial infarction
CAG: Coronary angiography
CT: Computed tomography
PFO: Patent foramen ovale
TIMI: Thrombolysis in Myocardial Infarction
UTUC: Upper tract urothelial carcinoma
